# NIVO-TIL: combination anti-PD-1 therapy and adoptive T-cell transfer in untreated metastatic melanoma: an exploratory open-label phase I trial

**DOI:** 10.2340/1651-226X.2024.40495

**Published:** 2024-11-07

**Authors:** Jean-Matthieu L’Orphelin, Ugo Lancien, Jean-Michel Nguyen, Francisco J. S. Coronilla, Soraya Saiagh, Julie Cassecuel, Lise Boussemart, Anne Dompmartin, Brigitte Dréno

**Affiliations:** a Department of Dermatology, Caen-Normandie University Hospital, Caen, France; b Interdisciplinary Research Unit for Cancer Prevention and Treatment, Université de Caen Normandie Inserm Anticipe, Normandie University, Research Building, Caen, France; c Department of Plastic Surgery, Nantes University Hospital, Nantes, France; d Nantes – Angers INSERM, Immunology and New Concepts in ImmunoTherapy, Nantes Université, INCIT, Nantes, France

**Keywords:** Melanoma, immunotherapy, anti-PD-1, TIL, adoptive cell transfer

## Abstract

**Background and purpose:**

In patients with metastatic melanoma who respond to anti-PD-1 therapy, the proliferation of intra-tumour CD8+ T cells is directly correlated with the clinical response, making tumour-infiltrating lymphocytes (TILs) a treatment of interest in combination with a PD-1 inhibitor, which is the undisputed gold standard in the management of metastatic melanoma. The aim of this trial was, therefore, to evaluate the safety and efficacy of sequential combination therapy consisting of nivolumab (a PD-1 inhibitor) and TILs adoptive T cells in patients with metastatic melanoma.

**Materials and methods:**

We performed an exploratory, prospective, single-centre, open-label, non-randomised, uncontrolled phase I/II study. We enrolled 10 previously untreated patients with advanced melanoma. The treatment regimen was neoadjuvant anti-PD-1 therapy followed by 2 injections of TILs and a second sequence of anti-PD-1 therapy.

**Results and interpretation:**

Among the four patients who received the autologous TILs + nivolumab combination, three (75%) achieved an objective response (two achieved a partial response [PR] at the end of the study, two achieved a complete response [CR]), and one achieved a CR at the end of the study. Among these three patients, one had a PR, and two had stable disease (SD) after the nivolumab course and before any TILs administration, reinforcing the importance of the tumour response after TILs injection. These responses were persistent, ranging from 9 months to 3.4 years.

## Introduction

The prognosis of patients with melanoma has been improved by immune checkpoint inhibitors (ICIs) [1] led by anti-PD-1 (programmed cell death-1) therapy. The superiority of ICIs has been confirmed by Ugurel et al. [2], revealing a benefit in overall survival (OS) compared with all other first-line curative metastatic treatments and confirming that ICIs are the gold standard in the management of metastatic melanoma. Despite these encouraging results, nearly 60% of patients will experience an initial lack of response or distant relapse after the initial response [3, 4]. Therefore, there is a need to strengthen the therapeutic arsenal to improve the clinical response [5].

The immune-activating mode of action of checkpoint inhibitors involves long-term tumour control and survival implications that are optimised when these drugs are administered prior to resection (neoadjuvant setting) and extend far beyond improved operability [6–8]. The neoadjuvant approach has never been as promising as it is currently, with exciting results from clinical trials such as SWOG 1801 [9] and NADINA [10].

A clinically relevant immune escape mechanism is the activation of the PD-1 receptor on tumour-infiltrating T lymphocytes [11]. By blocking the PD-1/PD-L1 interaction and restoring the effector function of PD-1+ T cells [12], anti-PD-1 therapy restores T lymphocyte cytotoxicity directed against antigens to effectively destroy tumour cells [5]. In metastatic melanoma patients who respond to anti-PD-1 therapy, the proliferation of intra-tumour CD8+ T cells is directly correlated with the clinical response [13].

Tumour-infiltrating lymphocytes (TILs) can recognise and kill tumour cells and could be a potential curative treatment with adoptive cell transfer (ACT) [14], further suggesting that TIL-ACT is an ultimate personalised treatment since a specific infusion product must be manufactured for each individual patient [5]. It consists of the outgrowth of tumour-resident T cells from tumour material, their expansion *ex vivo* and their transfer back into the same patient. TILs may be combined with a prior lymphodepletion regimen or administered alone, as done by Rohaan et al. [15]. ACT generates large numbers of tumour-reactive TILs, activates cells *ex vivo* and allows the patient’s immune system to prepare for the invasion of effector cells [16, 17].

The optimisation of ACT approaches involves anti-tumour T cells, which combines tumour specificity and reactivity, requires both functional improvement of the injected T cells and modulation of the tumour microenvironment, and enhances the recruitment of these T cells and their activation [18] while avoiding autoimmune side effects [19]. Nantes’ group has long focussed on the study of melanoma TIL. After observing the high degree of specificity and strong reactivity of some of these TILs for autologous tumour cells, we initially wished, similar to the Rosenberg group, to test the clinical efficacy of TILs transfer. For this purpose, they developed a culture method enabling both strong and systematic TILs expansion from small tumour fragments and the preservation, during this expansion phase, of TILs specificity for autologous tumour cells [20]. An initial phase I clinical trial using TILs produced by this method led to four regressions in six treated metastatic melanoma patients [21]. We thus wished to more precisely evaluate the clinical efficacy of these TILs. For this purpose, we conducted a randomised trial involving patients with melanoma at the locoregional invasion stage (AJCC stage III). The primary aim of this trial was to compare survival without relapse and overall survival of patients receiving either TILs and interleukin (IL)-2 together or IL-2 alone as adjuvant treatment after complete tumour excision. The results revealed that the infusion of autologous TILs significantly prolonged the relapse-free survival of a subgroup of patients bearing a single lymph node (LN) metastasis before treatment [19, 22–24].

The major limitation of ACT in stage IV melanoma is access to metastatic tissue to extract TILs and expand them *in vitro*. The two easiest locations are the skin and LNs. One of the escape mechanisms is mediated by regulatory T cells and the expression of inhibitory molecules such as PD-1 or CTLA-4 [25] by TILs. Nonetheless, the efficiency of ACT alone in the treatment of advanced metastatic melanoma patients remains a matter of debate. The absence of absolute efficacy of adoptive T cell therapy at the metastatic stage of melanoma could be explained by several mechanisms that inhibit the ability of T cells to eliminate antigen-expressing tumour cells *in vivo*. These tumour-specific T cells could be functionally deficient, anergic or unable to fully differentiate because the tumour environment could lack ‘danger signals’ or other innate immune stimuli, preventing a general inflammatory reaction [26, 27]. The immune inhibitory mechanism could be related to the tumour itself, with the expression of PD-L1/B7-H1 and indoleamine 2,3-dioxygenase (IDO), the release of cytokines such as TGF-β and IL10, and the loss of MHC class I or class II expression [28]. Another escape mechanism involves regulatory T cells and the expression of inhibitory molecules such as PD-1 or CTLA-4 by TILs [25, 29]. Several approaches could improve the clinical efficacy of ACT in melanoma patients, such as improving the level of immuno-depletion required to optimise the persistence of transferred cells and improving the clinical efficiency of ACT treatments. Indeed, a profound level of immuno-depletion is accompanied by serious toxicity and does not allow the spread of immune responses, which is suggested to be a determinant of the efficiency of some immunotherapy strategies. Nonetheless, the elimination of regulatory T cells in patients remains an important question, as do other regulatory mechanisms that could impair T-cell functions *in vivo*. Furthermore, the Rosenberg team examined PD-1 expression on TILs in metastatic melanoma lesions [11]. They showed that PD-1 is expressed by a greater number of TILs than T cells in normal tissue and peripheral blood in the same patient as well as healthy donors. Compared with PD-1- TILs, PD-1+ TILs displayed an exhausted phenotype and impaired effector function. Their study demonstrated that PD-1+ TILs exhibited diminished cytokine production in response to phorbol 12-myristate 13-actetate (PMA)/ionomycin, a potent T-cell stimulus that bypasses T cell receptor (TCR) signalling to activate diacylglycerol (DAG) and open calcium channels. They proposed that the diminished effector cytokine production by PD-1+ TILs, even in response to a potent stimulus, was reflected in their suppressed effector function compared with that of PD-1-TILs. Given that PD-1+ TILs display an impaired capacity to produce interferon-gamma (IFN-γ), an essential cytokine required for an effective anti-tumour immune response, their ability to destroy tumour cells is undermined and may lead to tumour immune escape.

For these reasons, TILs infusions were not preceded by non-myeloablative (NMA) lymphodepletion, and the number of cells infused by Nantes’ group was approximately tenfold lower [23]. Previous results concerning the use of ACT-TILs as an adjuvant therapy for stage III melanoma [30] allowed us to hypothesise that the efficacy of TILs in stage III melanoma may be directly related to the number of invading LNs and that tumour burden may impact the efficacy and/or *in vitro* expansion of tumour-specific T cells [31]. There is an association between the efficacy of TILs and the number of invading LNs [32], suggesting that a low tumour burden allows the curative effect of TILs. The clinical efficacy of such a therapeutic strategy could be further enhanced by the selection of highly reactive T cells for adoptive therapy via pretreatment with ICIs such as anti-PD-1 [19] to reduce the tumour burden and, thus, increase the presumed benefit of TILs treatment [23].

Therefore, we conducted this trial to evaluate the safety and efficacy of a sequential treatment defined as three courses of nivolumab (PD-1 inhibitor) first and then combined a PD-1 inhibitor and TILs adoptive T cells with IL-2 in patients with metastatic melanoma.

## Material and methods

### Design

We performed an exploratory, prospective, single-centre, open-label, non-randomised, uncontrolled phase I study. We enrolled 10 patients with advanced melanoma, and a protocol was provided for the inclusion of 11 people.

This research was conducted in accordance with good practices and received an initial favourable opinion on 13 October 2016 by the personal protection committee (CPP Ouest 6 Brest) that evaluated the protocol (Ref. RC 15_0247; registration number is N° EudraCT: n° 2015-005066-31). The biocollection has been registered under number DC-2011-1399 with the approval of CPP Ouest IV dated 08 November 2011.

### Inclusion and exclusion criteria

The inclusion criteria were as follows: patients over 18 years of age and weighing ≥ 40 kg and who provided informed consent; patients with stage IIIb, IIIc or IV metastatic melanoma (AJCC 6th edition) presenting at least two target lesions (LN relapse, in-transit nodule, unresectable skin metastases or visceral metastases other than bone or brain metastases), one of which was easily accessible, with a maximum of two lines of metastatic melanoma therapy; a negative pregnancy test for women of childbearing age, ECOG = 0 or 1; and Karnofsky score > 80%.

The non-inclusion criteria were bone or brain metastases; ocular melanoma; chemotherapy or radiotherapy within 4 weeks of baseline (6 weeks for nitrosourea and mitomycin C); contraindication to the use of vasopressors; a history or current manifestations of severe progressive heart disease; and patients previously treated with anti-PD-1, anti-PD-L1, anti-PD-L2, anti-CTLA4, or any other antibody or drug specifically acting on T-cell co-stimulation or immune checkpoints, except in the adjuvant or neoadjuvant setting. History of hypersensitivity to any product in the protocol, chronic autoimmune disease, inflammatory bowel disease, celiac disease or other chronic gastrointestinal diseases; presence of an active secondary cancer, with the exception of cervical cancer in situ or skin cancer other than previously treated melanoma; and patients requiring systemic treatment with > 10 mg prednisone equivalent per day or other immunosuppressive therapies within 14 days prior to nivolumab administration and adults under legal protection (guardianship, curatorship, or safeguard of justice).

The exclusion criteria were positive serologies for HIV1/2, p24 Ag, HTLV1, HTLV2, hepatitis B and C or syphilis.

### Treatment regimen

**Figure 1:**
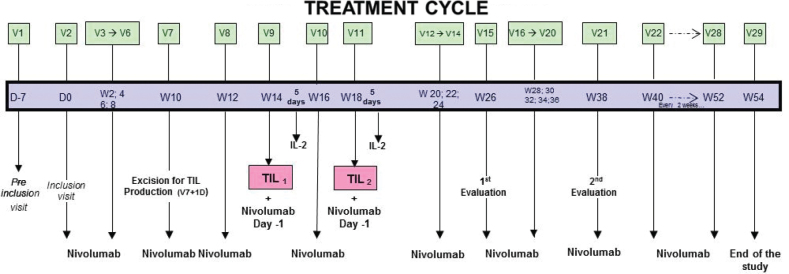
Treatment cycle from the initial inclusion visit to the end of the study.

The treatment regimen is detailed in Figure 1. The combined therapy of TILs was administered as follows:

- 1st step: Involves three courses of 3 mg/kg neoadjuvant anti-PD-1 (nivolumab) every 2 weeks to stimulate the cytotoxic activity of TILs present in the metastasis.
- 2nd step: Two injections of TILs were performed: the first occurred 30 ± 2 days after excision of the metastatic lesions, and the second occurred 30 ± 2 days after the first injection. An anti-PD-1 injection was performed between the two injections of TILs. TILs injections were systematically followed by injections of Proleukin^®^ (IL-2) 6 MUI I.V. per day for 5 days to promote the survival, multiplication and cytotoxic function of TILs *in vivo.* IL-2 treatment was started between 3 and 6 h after the end of the TILs injection.
- 3rd step: After TILs injection, a second sequence of anti-PD-1 at 3 mg/kg every 2 weeks was administered to complete 1 year of total treatment (from 052).

### TILs production procedure

The whole manufacturing process is provided in the supplementary material.

### Selected lymph nodes

Only single invading macroscopic LNs were selected for this trial. The size of each LN had to be sufficient to obtain expansion. From each invaded LN, 50% of the tissue was used for TILs expansion, 30% was used to obtain the melanoma cell line, and 20% was used for immunochemical analysis of the LN tissue from which the TILs were extracted.

### TILs production procedure

TILs were produced under ‘good manufacturing practices’ in the Cell and Gene Therapy manufacturing facility of Nantes University Hospital according to a previously described procedure [20, 21, 33]. TILs were extracted and expanded from the distant lesions of all patients included in this trial. TILs were minimally cultured for a short period of time with low-dose recombinant interleukin-2 (rIL2) [20, 33]. Short-term cultured TILs were isolated by culturing fragments of metastatic LN into 12-well tissue culture plates with X-VIVO 15 serum-free medium (BioWhittaker, Walkersville, MD, USA) containing low doses of rIL2 (150 U/mL) (Eurocetus, RueilMalmaison, France) and glutamine (1 mM, BioWhittaker) for 10–14 days. *Ex vivo* expanded TILs were derived as follows: 1.8 × 10 short-term cultured TILs were plated at 300 viable lymphocytes/well with irradiated feeder cells (allogeneic peripheral blood leukocytes [PBLs] and Epstein-Barr virus-infected B [B-EBV] cells) into U-bottom microplates in low-dose rIL2 medium (150 µL). PHA-L (phytohemagglutinin-L or leucoagglutinin) (Difco, Detroit, ML, USA) was added on Day 0 (1 µg/mL). Ten days later, lymphocytes were recovered from the culture plates, adjusted to 1 × 10 cells/mL in rIL2 medium and transferred into culture cell bags for an additional 10 days. The final TILs harvest was obtained by centrifuging, washing and suspending the TILs in 4% human serum albumin (LFB; Les Ulis, France). A second TILs expansion was performed within 1 month of the first, starting from cryopreserved short-term cultured TILs. Aliquots of TILs suspensions injected into patients were cryopreserved to study their tumour specificity, which was carried out later once the autologous tumour cell line had been established in culture. In 2007, following a request from the French Health Agency, the original culture medium used was replaced with X-VIVO™ Media (LonzaLevollois-Perret, France). Each patient received at least 1 billion TILs at each injection.

The total quantity of cells was determined. TILs were administered intravenously at 3 mL/min for a total duration of 3,065 min, corresponding to a total volume of 100,200 mL. IL2 was injected subcutaneously at a dose of 6 million UI between day 1 and day 5, and between day 8 and day 12. In the event of adverse effects (AEs), IL2 doses were interrupted and restarted after AEs had disappeared; if AEs reoccurred, IL2 treatment was definitively stopped.

### Establishment of melanoma cell lines

Fresh LNs with metastasis were minced into small tumour pieces and plated in a 24-well plate with 1.5 mL of RPMI (Roswell Park Memorial Institute) medium well supplemented with 10% foetal calf serum. The plates were incubated at 37°C in a humidified incubator with 5% CO, observed under a light microscope every week and sub-cultured when necessary.

### Cytokine production

The proportion of tumour-reactive TILs was determined through the measurement of the proportion of IFN-γ-secreting T cells within the TILs stimulated with the autologous melanoma cell line, as described previously [33]. For each cell line, a total of 500,000 cells per well of a 6-well plate were seeded in 3 mL of culture medium with or without 500 U/mL recombinant IFN-γ (Tebu, Le Perrayen Yvelines, France) in duplicate. After 48 h of incubation, the cells were washed, detached from the wells with PBS-EDTA (Lonza, Levallois, France), and processed for flow cytometry.

### Flow cytometry

Two millilitres of TILs cell suspension was collected in culture trays and transferred to a 15-mL tube. Then, the samples were transferred to Nantes University Hospital Immunology Laboratory for cytometry. Lymphocytes were gated according to their forward and size scatter characteristics and fluorescence-activated cell sorting (FACSC). Analysis was performed via BDFACS Diva software (BD Biosciences, San Jose, CA, USA), and the expression of several markers was explored (Table 2). Negative control assays were performed using a mouse monoclonal immunoglobulin G1 (IgG1) isotype control or a monoclonal immunoglobulin G2a (IgG2a) isotype control (Dako Cytomation).

**Table 1:** Patient characteristics.

	Patients						
	02	04	05	07	08	09	10
***Sex***	M	M	M	M	M	F	F
***Age***	74	80	56	57	67	73	61
***Primary***	LMM	UKN	ALM	UCL	UKN	MLM	SSM
***Breslow (mm)***	1.3	UKN	3.7	20	0.8	/	0.4
***Mutation***	0	0	0	NRAS Q61R	BRAF V600E	NRAS G13R	BRAFV600E
***Stage***	IVM1c	IVM1a	IVM1c	IVM1c	IVM1d	IVM1c	IIIc
***Target lesions***	2 pulmonary (right and left), 2 hepatic	3 right leg skin lesions	thoracic nodule, left iliofemoral adenopathy, left hilar	right upper lobe	right frontal and right adrenal	right paravertebral; right extra pleural; left adrenal; left hip; left kidney	cutaneous left leg
***Nontarget lesions***	left parotid lymph node	right foot skin and right leg skin	left inguinal adenopathy	lung, liver, skin	subcutaneous opposite left scapula	nasal cavity; peritoneal; hepatic; pre-colic	cutaneous left ankle, cutaneous left leg (*n* = 2), medial left leg
***Reason for exiting the study***	Death	End of the study	End of the study	Formalised TILs	No lesion for TILs excision	End of the study	End of the study

ALM: acrolentiginous melanoma; LMM: lentigo malignant melanoma; MLM: mucosal melanoma; SSM: superficial spreading melanoma; UKN: unknown primitive; UCL: unclassified melanoma.

Among the seven patients included, patients 02, 07 and 08 received PD-1 inhibitor treatment alone, whereas patients 04, 05, 09 and 10 received the combination of PD-1i+TIL.

**Table 2:** TILs phenotypes and cytotoxic activity determined via IHC using the streptavidin/peroxidase technique.

	Patient 04		Patient 05		Patient 09		Patient 10	
	TILs n°1	TILs n°2	TILs n°1	TILs n°2	TILs n°1	TILs n°2	TILs n°1	TILs n°2
*LT CD3+ (%)*	96.6	97.1	99	98.6	22.5		97.3	97.4
*Among CD3+: CD4+/CD8- (%)*	48.5	47.7	30.1	17.7	2.6		24.6	16.7
*Among CD3+: CD4+/CD8+ (%)*	2.8	1.8	1.7	2.5	0.9		9.4	17.5
*Among CD3+: CD4-/CD8- (%)*	2.6	1.5	22.8	34.3	2.6		1.2	1.4
*Among CD3+: CD4-/CD8+ (%)*	42.7	46.1	44.2	44.1	16.4		62.1	64.6
*LB (CD3-/CD19+) (%)*	0	0	0	0	0.1		0	0
*NK (CD3-/CD16+/56+) (%)*	2.2	1.9	0.8	0.9	75.5		2.2	2.4
*Cytotoxicity marker of NK: CD 107a+ INF γ+ (% of injected cells)*	31	44	15	24	11.5		12.9	
*Number of TILs injected (x10^9^)*	≤ 0.5	≤ 0.5	≤ 0.5	≤ 0.5	≤ 0.5		1≤ D≤20	
*Number of viable TILs (x10^9^)*	0.5	0.5	0.35	0.5	0.334		2.88	

TILs: tumour-infiltrating lymphocytes.

### Immunohistochemistry

Immunohistochemistry (IHC) was performed using the streptavidin/peroxidase technique [34]. The deep-frozen sections were incubated for 30 min at room temperature with the primary antibody. Different monoclonal antibodies, including CMH I and II, HLA-A2, CD54, Melan A, gp 100, pan-MAGE, BTLA, PD-1, PD-L-1, IDO, NY-ESO-1, CD3, CD8, CD4, TIM-3 and tyrosinase, were used to explore expression markers. Negative control assays were performed using a mouse monoclonal immunoglobulin G1 (IgG1) isotype control or a monoclonal immunoglobulin G2a (IgG2a) isotype control (DakoCytomation, DakoCytomation Denmark A/S ProdGBtionsvej 42, Glostrup, Denmark). The slides were read with a Leica microscope (magnification 25×). In each immune-stained serial section, the entire tumour area was evaluated. Each score was evaluated on a five-point scale as follows: absence of expression, weak expression (1%–25% of positive cells), moderate expression (26%–50%), intermediate expression (51%–75%) and strong expression (> 75%), corresponding to 0, 1, 2, 3 and 4, respectively.

### Outcomes

The primary objective was to evaluate the clinical and biological safety of adoptive T-cell transfer combined with anti-PD-1 therapy. The secondary objectives were to evaluate treatment efficacy, duration of clinical response, progression-free survival (PFS), and OS. All the subjects who received at least one injection of TILs were included in the efficacy analysis. PFS was defined as the time from random assignment in a clinical trial to disease progression or death from any cause.

All grade III-IV adverse events were classified and managed according to the common terminology criteria for adverse events (CTCAE) guidelines.

## Results

### Recruitment and analysis of the study population

The inclusion of the first patient occurred on 03 May 2018, and the date of the last visit or consultation of subjects included in the study was 18 October2022, which was considered the end date of the research. Ten patients, seven men and three women (sex ratio [M/F] of 2.3) with a mean age of 71 years, were included in the study. The following 10 patients signed a consent form and were included in the study:

- Three patients withdrew from the study before starting treatment with nivolumab (Patient 01 withdrew his consent; Patient 03 had a lung lesion that could not be biopsied to confirm melanoma; Patient 06 died because of rapid progression of his melanoma before the start of treatment).
- Seven patients received treatment (stage IIIC [*n* = 1], IV M1a [*n* = 1], IV M1c [*n* = 4] and IV M1d [*n* = 1]), four of whom were treated with at least one dose of TILs. All patients in this trial were naïve to any previous treatment, even though previous treatment was possible according to the inclusion criteria.

The details and characteristics of the patients are provided in Table 1.

### TILs production

**Figure 2:**
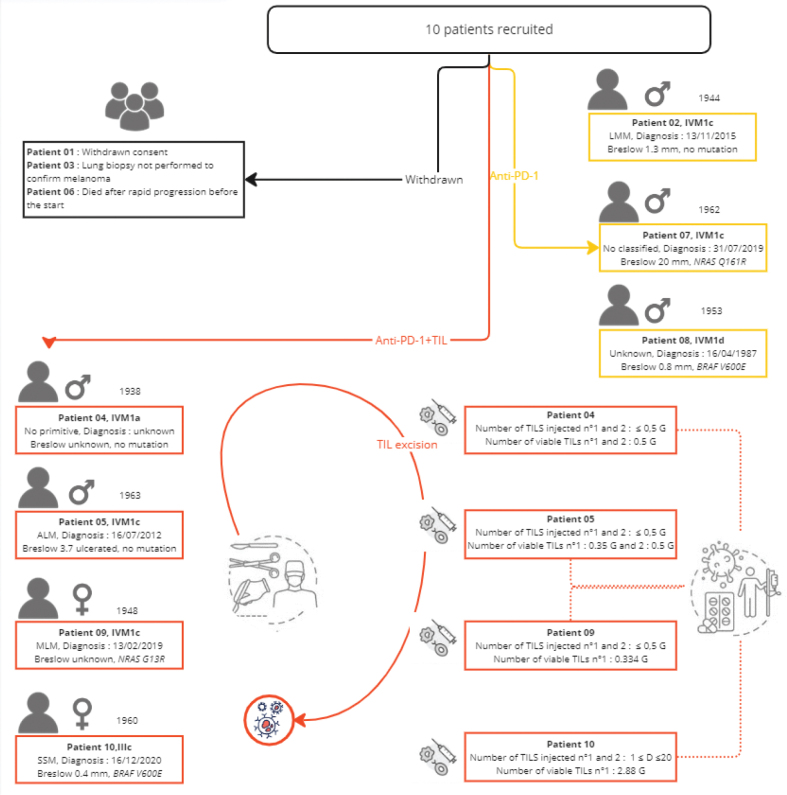
Individual outcomes of 10 recruited patients in the NIVO-TILs exploratory trial depicted on a timeline. PD: progressive disease; PR: partial response; CR: complete response; SD: stable disease; Star: disease; TIL: tumour-infiltrating lymphocyte.

*In vitro* TILs amplification is patient dependent, which implies variability in the number of TILs produced. The number and viability of injected TILs are detailed in Figure 2. Table 2 shows the phenotypes of the injected TILs. The two most important CD3+ subpopulations are CD4+ CD8- and cytotoxic CD4- CD8+ and CD 107a + INFγ+ cells. The dose of TILs chosen for injection was half the minimum dose we could obtain, that is 0.5 billion TILs.

### Efficacy assessment

The descriptive efficacy analysis focussed only on the four patients who received TILs + anti-PD-1 therapy, as one patient withdrew consent, one died rapidly, a biopsy of a distant lesion was not performed in one patient, and only neoadjuvant anti-PD-1 therapy was possible for three patients in the absence of LN involvement, allowing satisfactory production of TILs, as described in Figure 2. Three of the patients recruited did not benefit from TILs injection, one because of early death because of progression (Patient 2) and two others (Patients 07 and 08) because there was no tissue left for TILs production after the initial course of nivolumab monotherapy.

Among the seven patients included in this study, patients 2, 7 and 8 received only nivolumab, and patients 4, 5, 9 and 10 received the TILs + nivolumab combination corresponding to the study treatment evaluated. Patient 09 received a single infusion of TILs at visit V9 because of insufficient TILs production for the second infusion, as shown in Figure 2. At study visit V7 and after five injections of nivolumab, only patients who experienced progression, partial response (PR) or stable disease (SD) were eligible for excision of a tumour accessible for TILs production. Patients with a complete response (CR) to nivolumab at visit V7 did not receive TILs and continued on nivolumab alone. For the seven patients treated, the mean PFS was 615 days.

All four patients treated with TILs + anti-PD-1 therapy completed the full treatment sequence up to visit V29 (month 12). For these four patients, the clinical response at the end of the study was one CR, two PRs and one disease progression for patients 10, 04, 09 and 05, respectively.

### Efficacy assessment

After the end-of-study visit at M12, the median follow-up was 560 days [274;1,240], with a persistent CR for three patients. Clinical data showing individual results and follow-up data after the end of the trial are summarised in Figure 2. Patient 04 presented with a PR at the end-of-study visit and showed persistent CR at the last visit (i.e. 1,240 days after the end-of-study visit). Patient 09 presented initial PR and CR 8 months after the end of the study, which persisted after the end of the study (i.e. 560 days). Patient 10 was in CR at the end-of-study visit, which has been persistent since then, with a 274-day decline.

**Figure 3:**
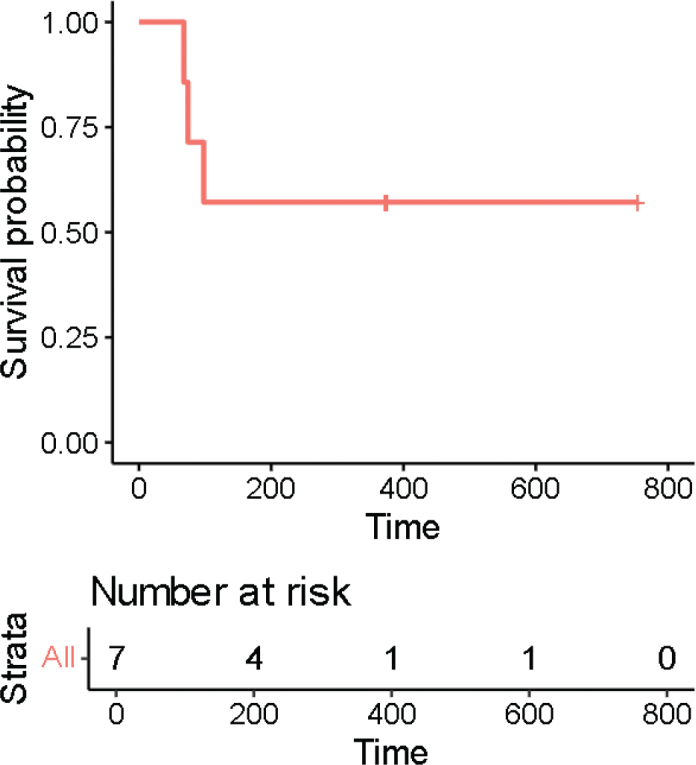
Kaplan-Meier curve for progression-free survival.

The mean PFS was 615 days, and the individual PFS rates were 77, 1630, 280, 86, 1284, 271 and 677 days for patients 2, 4, 5, 7, 8, 9 and 10, respectively. As three patients did not experience any progression, PFS was calculated from the date of the last visit. The PFS data are illustrated in Figure 3 via a Kaplan-Meier curve.

### Adverse events

Over the course of the study, five serious AEs (SAEs) occurred, 4 of which were considered unrelated to TIL, IL-2 or anti-PD-1:

- Patient 02: diagnosed with an ‘altered general condition’ and was discharged on 02 July 2018 with an adaptation of his analgesic treatment. A CT scan obtained on 04 July 2018 revealed that the tumour had progressed. He died on 16 July 2018 in the context of melanoma-related deterioration in general condition.
- Patient 04: ‘basal cell carcinoma’ was identified on the skin biopsy of a right scapular lesion performed at visit 8.
- Patient 05: ‘Haemorrhagic stroke’ was diagnosed on 06 May 2020 following treatment with nivolumab since 31 January 2019 and TIL/Proleukin^®^ on 05 September 2019 and 06 May 2019. The stroke corresponded to bleeding from a metastasis.
- Patient 09: ‘Infiltrating carcinoma of the right breast’ was diagnosed on 16 November 2021 following treatment with nivolumab since 30 November 2020, and a TILs injection was administered on 17 March 2021, followed by protocol injections of Proleukin^®^. The investigator and sponsor considered that there was no reasonable possibility that the event was related to TIL/Proleukin or nivolumab but rather to the patient’s advanced age.

Only one SAE, ‘myocardial infarction’, was reported for Patient 06, a 74-year-old man with known cardiovascular risk factors (hypertension, overweight, diabetes, hypercholesterolemia). He received the first administration of nivolumab on 15 July 2019 and was admitted to the hospital on 20 July 2019 for dyspnoea on exertion, palpitations and suspected pulmonary embolism, enabling a diagnosis of myocardial infarction complicated with renal failure and tachycardia. The patient died on 27 July 2019, and the investigator and sponsor considered that there was a reasonable possibility that the event was related solely to nivolumab (TILs) and Proleukin^®^ not having been administered prior to the occurrence.

The safety profile was satisfactory (80.1% were non-severe AEs). Two non-severe AEs were reported, namely, asthenia and coxarthrosis, which were initially moderate and worsened, with sequelae persisting at the end of the follow-up of this clinical trial.

Only one non-serious AE (hypotension) occurred with TILs treatment on the day of administration, and 11.6% of non-severe AEs were related to IL-2 in four patients. Details are provided in Table 3. AEs were attributed either to IL2 or anti-PD-1 therapy or to the combination of the two agents, according to the investigator’s pharmacovigilance data. With respect to the TILs alone, except for rare episodes of fever during IV injection, no serious AEs associated with TILs administration have been found in our department, which is consistent with the literature. The majority of non-serious AEs were possibly related to nivolumab (45.2%).

**Table 3:** Causality of nonserious adverse events: number of events attributable to IL-2, anti-PD-1 and TILs according to pharmacovigilance data reported by the investigator during the trial.

	IL-2	PD-1 inhibitor	TIL
**Blood and lymphatic**	1	5	0
**Cardiac**	0	1	0
**Endocrine**	0	6	0
**Eye**	0	1	0
**Gastrointestinal**	1	4	0
**General disorders**	7	9	0
**Hepatobiliary**	1	1	0
**Infections and infestations**	0	2	0
**Investigations**	2	16	0
**Metabolism and nutrition**	0	1	0
**Musculoskeletal and connective tissue disorders**	2	3	0
**Nervous system**	1	3	0
**Psychiatric**	0	1	0
**Renal and urinary**	0	0	0
**Respiratory and thoracic**	0	3	0
**Skin and subcutaneous tissue**	2	10	0
**Vascular**	0	0	1

TIL: tumour-infiltrating lymphocyte.

The non-severe AEs still in progress at the end of patient follow-up mainly involved chronic or slowly evolving manifestations (vitiligo, coxarthrosis, reflux), but signs persisted at the time of death or at the end of patient follow-up. Over the duration of the study, five serious AEs corresponding to five CTCAE grade three events were reported: four severe CTCAE grade three AEs unrelated to treatment and one CTCAE grade three AE related to treatment.

Concerning the four grade 3 severe CTCAEs unrelated to TILs or Proleukin^®^ (IL-2), nivolumab has the following characteristics: ‘alteration of general condition’, ‘basal cell carcinoma’, and ‘haemorrhagic stroke’ regressive with minimal sequelae such as psychomotor slowing and ‘infiltrating carcinoma of the right breast’. One CTCAE grade 3 myocardial infarction was considered potentially related solely to nivolumab (TILs and Proleukin^®^ not having been administered prior to the infarction). Treatment included a stent and appropriate drug therapy. The patient developed acute renal failure and tachycardia, which led to palliative care being initiated as the patient was at an advanced metastatic stage and then died.

## Discussion

This exploratory study demonstrated that the adoptive transfer approach of TILs in combination with anti-PD-1 therapy is well tolerated by patients and does not induce more AEs than those reported in the literature for patients treated with ICIs. Our approach is quite similar to the neoadjuvant approach with anti-PD-1 alone [9], which has already been proven to be effective in treating melanoma. Despite the small number of patients included in this study, the results show that the adoptive transfer approach of TILs combined with anti-PD-1 therapy is well tolerated by patients and does not induce more adverse events or autoimmune reactions than those reported in the literature in patients treated with anti-PD-1 immunotherapy alone or combined with anti-CTLA4 therapy. In the intention-to-treat population (*n* = 7), the best overall response was five CRs and two PDs. The mean PFS was 615 [77;1,630], but three patients did not have any progression, and the date used was the date of the last visit, which minimised PFS. Among the four patients who received the autologous TILs + nivolumab combination, three (75%) achieved an objective response (two achieved a PR at the end of the study, and two achieved a CR), and one achieved a CR. However, these good results must be cautiously considered in view of the small cohort of this exploratory trial, especially as only four patients received the combination of a PD-1 inhibitor and TILs. Furthermore, patients with minimal tumour burden, such as those treated in a neoadjuvant setting, are likely to have a particularly high chance of response. The frequency of complete pathological response to neoadjuvant PD-1 inhibition may be as high as 21% [9] and this figure was revised upwards at ASCO 2024 update. The initial patients who were non-responsive at the first evaluation did not necessarily indicate that the following response was because of the TILs treatment. A reasonable way to substantiate the claim that there indeed is an additional effect of TILs therapy would be to evaluate the degree of pathological response of the excised lesion. If patients with SD indeed had a pathological non-response (>50% viable tumour) and later developed a response upon TILs injection, it would be reasonable to state that TILs treatment had a contributory effect. In the absence of an available pathological response, more widely recruiting trials will be necessary.

Patient 09 received 75% NK cells, and patient 05 received 22%–34% CD3+/CD4-/CD8- cells. The outcome of ICI therapy in cancer patients has been linked to the quality and magnitude of T cell, NK cell, and, more recently, B-cell responses within the tumour microenvironment [35]. High expression of IFN-γ in human tumour-infiltrating NK cells and an enriched NK-IL18-IFN-γ signature in solid tumours correlated with increased overall patient survival. Thus, the inhibition of HIF-1α unleashes NK cell anti-tumour activity and could be exploited for cancer therapy [36]. Among these three patients, one had a PR, and two had SD after neoadjuvant nivolumab, suggesting the value of the tumour response after TILs injection, especially since one of the CRs was obtained in an NRAS-mutant MLM, which is a melanoma known to be aggressive [36]. Another acellular melanoma (ALM) is known to be a poor responder to immunotherapy, whereas we report a good response to the combination of TILs + anti-PD-1 therapy with a long follow-up without relapse. This is even more remarkable given that ALMs and mucosal melanomas (MLMs) are no-cumulative solar damage (CSD) melanomas, which are known to respond poorly to immunotherapy [37, 38]. The response was durable, ranging from 9 months to 3.4 years, which is greater than that reported by Zhao et al. [1], who reported that CR was achieved approximately 4 months after the initiation of TILs + nivolumab and was still complete 1 year after the cessation of treatment. With respect to protocols, TILs are often combined with prior lymphodepletion regimens [17, 39], whereas we did not precede TILs infusion by lymphodepletion on the basis of Nantes’ previous experience [22, 30, 32].

According to the literature, a phase II study [40] showed that adoptive transfer of autologous TILs in patients with unresectable melanoma refractory to anti-PD-1 therapy induced objective clinical responses in 36% of patients (2 CR) and that the majority of the responses were durable, with a median duration of response not reached. A phase III trial [15] demonstrated the superiority of TILs over ipilimumab (anti-CTLA-4) in patients with PD-1-resistant melanoma, with an improvement in median PFS from 3.1 to 7.2 months and median OS from 18.9 to 25.8 months. Like Sarnaik et al. [40] and Rohaan et al. [15], our study validates the role of TILs therapy in the therapeutic arsenal against metastatic melanoma as a second-line therapy after no response or PR to ICIs. Rohan et al. [15] used many TILs with a median size of 40 × 10^9^ cells, and we used no chemo-depletion and a lower dose of Il-2. Since TILs + nivolumab + IL-2 treatment will be administered for the first time to humans, TILs dose escalation will be performed. This escalation will not be performed to find a toxic dose limit since TILs are autologous cells but rather to ensure that the combined treatment (TILs + IL-2 + nivolumab) does not cause severe autoimmunity pathologies, justifying lower doses than those commonly administered. This cohort was not used to find a toxic dose limit for TILs, as TILs are autologous cells, but rather to ensure that the combined treatment (TILs + IL-2 + nivolumab) did not cause severe autoimmune pathologies. Depending on the patient, between 1 and 20 billion TILs can be obtained per production in our trial, as *in vitro* TILs amplification is patient dependent, which implies variability in the amount of TILs produced. The dose of TILs chosen was half the minimum dose we could obtain, that is 0.5 billion TILs.

The optimisation of the adoptive transfer of anti-tumour T cells requires both functional improvement of the injected T cells and modulation of the tumour microenvironment, which favour the recruitment of these T cells and their activation. Marotte et al. [18] demonstrated the safety and superiority of a combination of adoptive transfer of PD-1-deficient T cells and targeted alpha therapy (TAT) targeting PD-L1 to control the growth of melanoma tumours in immunodeficient laboratory mice. Thus, they provide the first proof-of-concept of the efficacy of combination therapy using TAT, ACT and genomic editing of IC-encoding genes. Here, we optimised the adoptive transfer of anti-tumour T cells by extracting TILs previously stimulated with both anti-PD1 antibody and the antigen expressed by the tumour cells.

Shi et al. [41] reported that ACT combined with ICI confers durable anti-tumour responses that depend on CD8+ T-cell-intrinsic expression of ICOS (inducible T-cell costimulatory, a T-cell-specific cell surface molecule).

Another method [14] is to enrich tumour-reactive T cells from a heterogeneous TILs population, the CD8+ PD-1+ 4-1BB+ TILs population, followed by direct reprogramming into induced pluripotent stem cells (iPSCs), enabling them to be enhanced for more potent ACT. Dréno et al. [19] reported that CD8+ T cells, defined by the co-expression of PD-1 and TIGIT, are associated with the efficacy of anti-PD-1 immunotherapy in metastatic melanoma patients. They reported that CD8+/PD-1+/TIGIT+ cells, named DPOS T cells, are associated with increased OS.

## Conclusion

Our preliminary data support the hypothesis that combined therapy with TILs + nivolumab is promising in patients with metastatic melanoma without prior lymphodepletion. Since our exploratory open-label phase I trial enrolled few patients, it needs to be tested in phase III trials. We do not report the emergence of new safety signals and the safety profile was satisfactory. We also need to consider the integration of this treatment as a neoadjuvant for stage III melanoma with the following design: anti-PD-1 therapy in the neoadjuvant setting followed by surgery and a combination of TILs + anti-PD-1 therapy for non-CR patients.

## Author contributions

Conceptualisation: Brigitte Dreno; Methodology: J-Michel Nguyen; Formal analysis and investigation: Jean-Matthieu L’Orphelin, Ugo Lancien, Jean-Michel Nguyen, Francisco Javier, Saenz Coronilla, Soraya Saiagh, Julie Cassecuel, Lise Boussemart, Brigitte Dreno; Writing – original draft preparation: Jean-Matthieu L’Orphelin; Writing – review and editing: Jean-Matthieu L’Orphelin; Lise Boussemart, Anne Dompmartin, Brigitte Dreno; Funding acquisition: Brigitte Dreno; Resources: Brigitte Dreno; Supervision: Anne Dompmartin, Brigitte Dreno.

## Data Availability

The datasets generated during and/or analysed during the current study are available in the research centre of Nantes, Immunology and New Concepts in ImmunoTherapy.

## Clinical trial registration

Protocol registration number is Ref. RC 15_0247; registration number is N° EudraCT: n° 2015-005066-31. The biocollection has been registered under number DC-2011-1399 with the approval of CPP Ouest IV dated 08/11/2011.
